# Sequencing, assembly, annotation, and gene expression: novel insights into browning-resistant* Luffa cylindrica*

**DOI:** 10.7717/peerj.9661

**Published:** 2020-08-10

**Authors:** Ya-Hui Wang, Xiao-Hong Liu, Rong-Rong Zhang, Zhi-Ming Yan, Ai-Sheng Xiong, Xiao-Jun Su

**Affiliations:** 1 Institute of Vegetable Crops, Jiangsu Key Laboratory for Horticultural Crop Genetic Improvement, Jiangsu Academy of Agricultural Sciences, Nanjing, China; 2 State Key Laboratory of Crop Genetics and Germplasm Enhancement, Ministry of Agriculture and Rural Affairs Key Laboratory of Biology and Germplasm Enhancement of Horticultural Crops in East China, College of Horticulture, Nanjing Agricultural University, Nanjing, China; 3 Jiangsu Vocational College of Agriculture and Forestry, Jurong, China

**Keywords:** Expression, *Luffa*, Polyphenol oxidase, Transcriptome, Browning

## Abstract

*Luffa* is a kind of melon crop widely cultivated in temperate regions worldwide. Browning is one of the serious factors affecting the quality of* Luffa*. Therefore, the molecular mechanism of *Luffa* browning is of great significance to study. However, the molecular diversity of *Luffa* cultivars with different browning-resistant abilities has not been well elucidated. In our study, we used high-throughput sequencing to determine the transcriptome of two* Luffa cylindrica* cultivars ‘2D-2’ and ‘35D-7’. A total of 115,099 unigenes were clustered, of which 22,607 were differentially expression genes (DEGs). Of these DEGs, 65 encoding polyphenol oxidase, peroxidase, or ascorbate peroxidase were further analyzed. The quantitative real-time PCR (RT-qPCR) data indicated that the expression levels of the *LcPPO* gene (Accession No.: Cluster-21832.13892) was significantly higher in ‘35D-7’ compared with that in ‘2D-2’. Several *POD* genes (Accession No.: Cluster-21832.19847, Cluster-21832.30619 and Cluster-48491.2) were also upregulated. Analysis of the plantTFDB database indicated that some transcription factors such as WRKY gene family may also participate in the regulation of *Luffa* browning. The results indicated that the divergence of genes expression related to enzymatic reaction may lead to the different browning resistances of* Luffa*. Our study will provide a theoretical basis for breeding of browning-resistant *Luffa*.

## Introduction

*Luffa* (*Luffa cylindrica*), belongs to *Luffa* Mill. of Cucurbitaceae, is an annual herbaceous climbing plant (Oboh & Aluyor, 2009). *Luffa* is native to India and tropical southern Asia and is mainly distributed in Asia, Oceania, Africa, and America. *Luffa* has eight species worldwide. China mainly cultivates *Luffa cylindrica* Roem. and *Luffa acutangula* Roxb. (Oboh & Aluyor, 2009; Xu et al., 2018). Compared with other Cucurbitaceae plants, the most remarkable characteristic of *Luffa* is the abundant reticular fibers composed of vascular bundles within fruit. With its peel, seeds and pulp removed, *Luffa* is considered as a traditional Chinese medicine, *Luffa* sponge (Asma & Muhammad, 2013). *Luffa* contains large amounts of vitamin B, vitamin C, saponins, citrulline, and other nutrients. Therefore, *Luffa* has effects on scurvy prevention, antivirus, cosmetology and so on (Mazali & Alves, 2005). Since *Luffa* was introduced into China during the Song Dynasty, it has been widely cultivated in China due to its strong adaptability, and a variety of local cultivars have gradually been formed (Su et al., 2010). However, the skin, pulp, and juice of *Luffa* fruits are prone to browning during the transportation and processing; browning seriously affects the nutritional value and flavor of the fruit, leading to the decline in the commercial quality of *Luffa* products (Friedman, 1991; Vamosvigyazo, 1995). Therefore, cultivating browning-resistant *Luffa* germplasm resources is of great significance.

Browning is a common discoloration phenomenon and a beneficial step in food processing, such as the productions of soy sauce, bread, black tea and coffee, etc. (Okada, Katsura & Kobayashi, 2004; Purlis & Salvadori, 2007; Troup et al., 2015; Lee & Knag, 2017). However, browning can affect the flavor and reduce the nutritional value of most fruits and vegetables and is therefore considered the second largest cause of quality loss (Lim, Cheun & Wong, 2019). The two main types of browning are as follows: enzymatic browning, which is the result of polyphenol oxidase (PPO) that catalyzes the formation of quinone and its polymer; and non-enzymatic browning, which involves Maillard reaction and caramelization (Buera et al., 1987; Zamora & Hidalgo, 2005). The browning of *Luffa* is mainly due to enzymatic browning. When *Luffa* is stimulated by collision, friction, or heating, the content of active oxygen increases, thereby damaging the cellular structure of *Luffa*. The compartmental distribution of enzymes and phenolic substances will be broken and lead to the formation of quinones (Xu et al., 2018). Numerous enzymes involved in this process are polyphenol oxidase, ascorbate peroxidase (APX), and peroxidase (POD), etc. (Nicolas et al., 1994; Jiang et al., 2016). Research on the mechanism of browning has been carried out in several species (Ali et al., 2016; Liu et al., 2019). Jiang (2000) found that anthocyanins, polyphenol oxidase, and phenols participated in the lychee pericarp browning. Studies in potato showed that the antisense expression of *PPO* genes can inhibit the browning of potato tubers (Bachem et al., 1994). Browning in fresh eggplant is related to the phenolic content and specific activity of PPO (Mishra, Gautam & Sharma, 2013). These results provide a reference for the exploration of the mechanism of *Luffa* browning and make up for the lack of related studies.

The development of transcriptome sequencing techniques has led to identification of novel transcripts, splice isoforms, and expression differences in numerous species (Wu et al., 2014; Wang et al., 2015). A large amount of sequences and plant molecular information have been revealed using omics analysis (Li et al., 2018; Li et al., 2020), but the application of such research in *Luffa* is still lacking. In the present study, two *Luffa* cultivars were used. ‘2D-2’ is a kind of browning-resistant cultivar and ‘35D-7’ is a cultivar prone to browning. Both of the two cultivars are self-bred lines which were originated from ‘Early Rou *Luffa*’ and ‘Short Stick-like Rou *Luffa*’, respectively. Transcriptome analysis was conducted to investigate the divergence between the two *Luffa* cultivars with browning-resistant or -sensitive characteristics. Differentially expression genes (DEGs) related to *Luffa* browning were identified. Expression levels of related genes were also validated and analyzed. Our results can provide potential insights into the molecular regulation of *Luffa* browning and a basis for cultivating improved *Luffa* germplasm with high browning resistance.

## Materials & Methods

### Plant materials and sample collection

The seeds of the two *Luffa* cultivars, ‘2D-2’ and ‘35D-7’, were preserved in the Institute of Vegetable Crops, Jiangsu Academy of Agricultural Sciences and cultivated in the farm of Luhe Base (118.62°N, 32.49°E, Nanjing, China). The field was approved for using by Jiangsu Academy of Agricultural Sciences (Contract No.: SC2018001). The fruits of each cultivar were collected after 10 days after pollination. The fruit pulps near the end of the stalk that are prone to browning were sampled for subsequent experiments. Samples were collected from three individual fruits. The samples were immersed in liquid nitrogen immediately and stored at −80 °C.

### RNA extraction and quality control

The total RNA of each sample was extracted according to the instructions of the plant RNA simple total RNA (Tiangen, Beijing, China). Agarose gel electrophoresis and Agilent Bioanalyzer 2100 (Agilent, China) was used to detect the integrity of the RNA samples. RNA concentration and purity were measured by NanoPhotometer (Implen, Germany) and Qubit 2.0 Fluorometer (ThermoFisher, USA).

### Construction of cDNA library and sequencing

Construction of cDNA library was conducted in accordance with the instructions of NEBNext Ultra Directional RNA Library Prep Kit for Illumina (NEB, Beijing, China). Ribosomal RNA was removed from total RNA to obtain the mRNA of the samples. Fragmentation buffer was added to break RNA into small fragments, which were used for the synthesis of the first-strand cDNA. Buffer, dNTPs (dUTP, dATP, dGTP, and dCTP), and DNA polymerase I were utilized to synthesize the second chain. The obtained double-stranded cDNA was purified by AMPure XP beads. After terminal repairing, A-tail adding, indexing adapter ligation, and fragment size selection, PCR amplification was conducted to enrich the short fragments for construction of the final cDNA library. The cDNA library was further quantified and its insert size was detected by Agilent 2100 to ensure that its quality could achieve the standard for sequencing. Sequencing was performed on the NovaSeq 6000 Sequencing System (Illumina, California, USA). Paired-end (150bp) sequencing was set as sequencing strategy.

### Data filtering and de novo assembly

Adapter-related reads, low-quality reads, and reads with relatively high number of base ‘N’ were filtered to ensure the accuracy of subsequent analysis. Clean reads after raw data filtering, error rate checking, and GC content distribution checking were used for subsequent transcript assembly. Trinity 2.6.6 was used to assemble the clean reads to obtain the reference sequence (Grabherr et al., 2013). The longest cluster sequence obtained by Corset hierarchical clustering (https://code.google.com/p/corset-project/) was used as unigene for subsequent analysis.

### Unigene function annotation

BLAST software was used to search the unigene sequence against Kyoto Encyclopedia of Genes and Genomes (KEGG) (Minoru et al., 2007), NCBI non-redundant protein (NR) (Pruitt, Tatiana & Maglott, 2005), Swiss-Prot, Gene Ontology (GO) (Mi et al., 2019), Clusters of Orthologous Groups of proteins (COG) (Natale et al., 2000), and TrEMBL database (Boeckmann et al., 2003) (*E*-value ≤ 1E-5). The predicted amino acid sequences of the best aligning unigenes were transferred and searched by Pfam database using HMMER software to obtain the annotation information of the unigenes (El-Gebali et al., 2019). BLASTX was used for further functional categorization with the *E*-value threshold of 1E-5.

### Identification of DEGs

DESeq2 was utilized to analyze the differential expression among samples. Non-standardized reads were counted by featureCounts. Multiple hypothesis testing of *P*-value was conducted based on Benjamini–Hochberg method. The transcription abundance of the samples was expressed by fragments per kilobase of transcript per million fragments mapped (FPKM). The rationale of the DEGs was set as follows: —log2fold change—≥1 and the false discovery rate (FDR) < 0.05. The function of the DEGs was annotated by KEGG, GO, and COG database, and DEGs related to browning were identified according to the annotation information. Transcription factor annotation was identified by iTAK version 1.7 software in reference of plantTFDB (Jin et al., 2017).

### Validation of the DEGs through quantitative real-time PCR (RT-qPCR)

Primer Premier 6.0 was used to design the specific primers of the DEGs. The primer sequences are listed in Table 1. *Lc18SRNA* was selected as reference gene (Singh et al., 1998). RT-qPCR was performed on Bio-Rad CFX96 PCR platform (Bio-Rad, USA) using Hieff qPCR SYBR Green Master Mix (Yeason, Shanghai, China).The reaction mixture contains 10 µL of enzyme, 2 µL of diluted cDNA, 0.4 µL of forward or reverse primer, and sterile deionized water was used to replenish the volume to 20 µL. The reaction procedure was set as follows: 95 °C for 5 min; 95 °C for 10 s and 60 °C for 30 s, 40 cycles in total. A melting curve was established by increasing from 65 to 95 °C to verify the amplification specificity. The obtained cycle threshold values were calculated using Pfaffl method (2001). Three independent biological replicates were set for each sample. Significance analysis was carried out in Microsoft Excel 2016 according to the method of one-way ANOVA.

**Table 1:** Primers used for RT-qPCR.

**Gene symbol**	**Description**	**Forward primer sequence (5′to 3′)**	**Reverse primer sequence (5′to 3′)**
Cluster-21832.13892	polyphenol oxidase	CGAATGTTCACTGTGCCTAT	CTTCGGAGCGTCGTAGT
Cluster-21832.19847	peroxidase	CAACATTGTCCGCCGTGAG	TCTGCTGTCTCTTCTTCCGTATA
Cluster-21832.30619		ACTGCTTCGTCAATGGATGTG	CTGTTGGCTATTCTGCTGTCTT
Cluster-21832.38395		GGTGGCTCTGCTTCTTATTACATA	TTGGTCTCCCTCTCTTCTTCTTT
Cluster-23349.0		GCATCACAAGAGCCAGGAAG	GTGGTCGGAGCATATCTATCAAC
Cluster-2660.0		GAGCGAGAGTGAAGCCAATC	CACCTGAGAGCACAACAACAT
Cluster-48491.2		GGTGAAGTGTACGGTGATGATG	GAATCTATCTGCCTCTATCCTCCT
Cluster-5215.0		GCTTCCACCAACATACACTCC	GCCGCCTACCAGAATCACT
Cluster-19973.6	ascorbate peroxidase	ACGGATGCTAACAACGAGGAT	CTGAACTGAAGCGGATGGATG
Cluster-21832.12599		GCTCCTGAAGACTACATTCT	GACCAGCATTAGCACCAT
Cluster-21832.17816		CTGCTGTTAGTGAGGAGTAT	CAATAGCCTAATAGCGATGTC
Cluster-21832.12605		GCCAACTGATGCTGCTCTC	GAATCTTCTGCTTCATTGAGTCTG
Lc18SRNA	reference gene	GTGTTCTTCGGAATGACTGG	ATCGTTTACGGCATGGACTA

## Results

### Sequencing and assembly of *Luffa*

**Figure 1:**
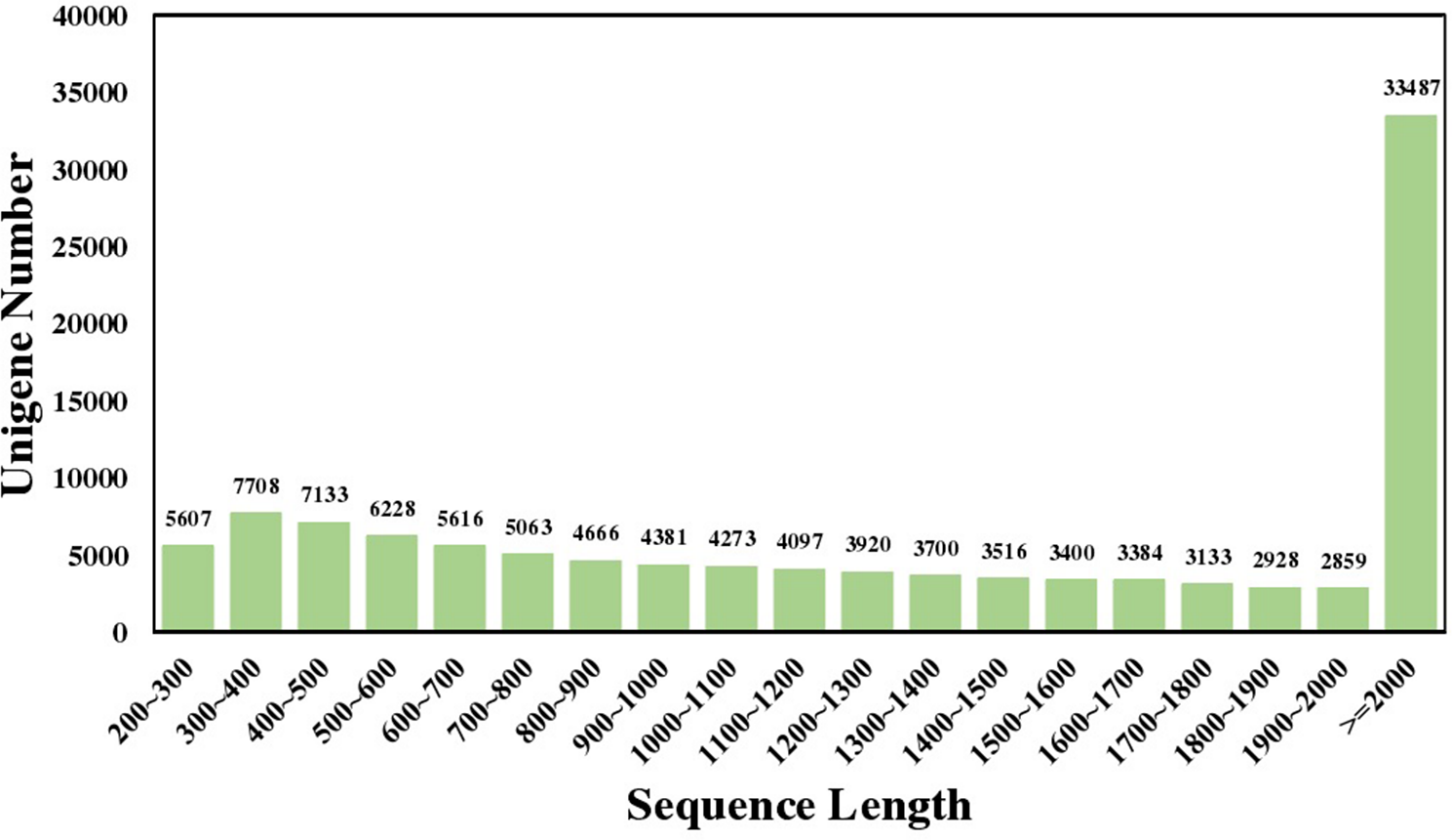
Sequence length distribution of the *Luffa* unigene library.

A total of 67,969,672 and 59,573,164 raw reads were detected by sequencing the two samples, ‘2D-2’ and ‘35D-7’, respectively. The sequencing raw data can be found in the Sequence Read Archive (SRA) database (Accession No.: SRP252794). Assembly sequences has been deposited at TSA database under the Accession GILZ00000000. After data filtering, 9.67 G and 8.49 G clean high-quality reads were obtained. The percentage of Q20 and Q30 of ‘2D-2’ and ‘35D-7’ were 98.53% and 95.3%, 98.52% and 95.3%, respectively. The GC percentage in high-quality reads were 44.12% and 44.74%, respectively. The number of the contigs found by assembling the sequences according to Trinity was 144,048 with a mean length of 1,311 bp and an N50 length of 2,160 bp. After clustering, the longest clusters were identified as unigenes, and the total number of unigenes was 115,099 and the mean length and N50 length was 1,573 bp and 2,235 bp, respectively. The length distribution of the unigenes is shown in Fig. 1. Most of the unigenes were over 2,000 bp in length (33,487, 29.09%), then followed by 300 to 500 bp (7,708, 6.70%).

### Functional annotation and classification

All unigenes were annotated against seven databases (KEGG, NR, SwissProt, TrEMBL, KOG, GO and Pram), and the detailed numbers of unigenes are listed in Table 2 (Table S1). The NR database annotated most of the 115,099 unigenes (99,246, 80.14%). The GO annotation classified the unigenes according to three categories of GO (biological process, cellular component, and molecular function), the results are displayed in Fig. S1. A total of 78,549 unigenes were branched into 58 function groups.

**Table 2:** Summary of annotation of *Luffa* unigenes.

**Annotation database**	**Annotated number**	**Proportion of all unigenes (%)**
KEGG	73,507	63.86
NR	92,246	80.14
SwissProt	66,525	57.8
TrEMBL	91,475	79.48
KOG	57,412	49.88
GO	78,549	68.24
Pfam	70,026	60.84
All Annotated	92,524	80.39

**Figure 2:**
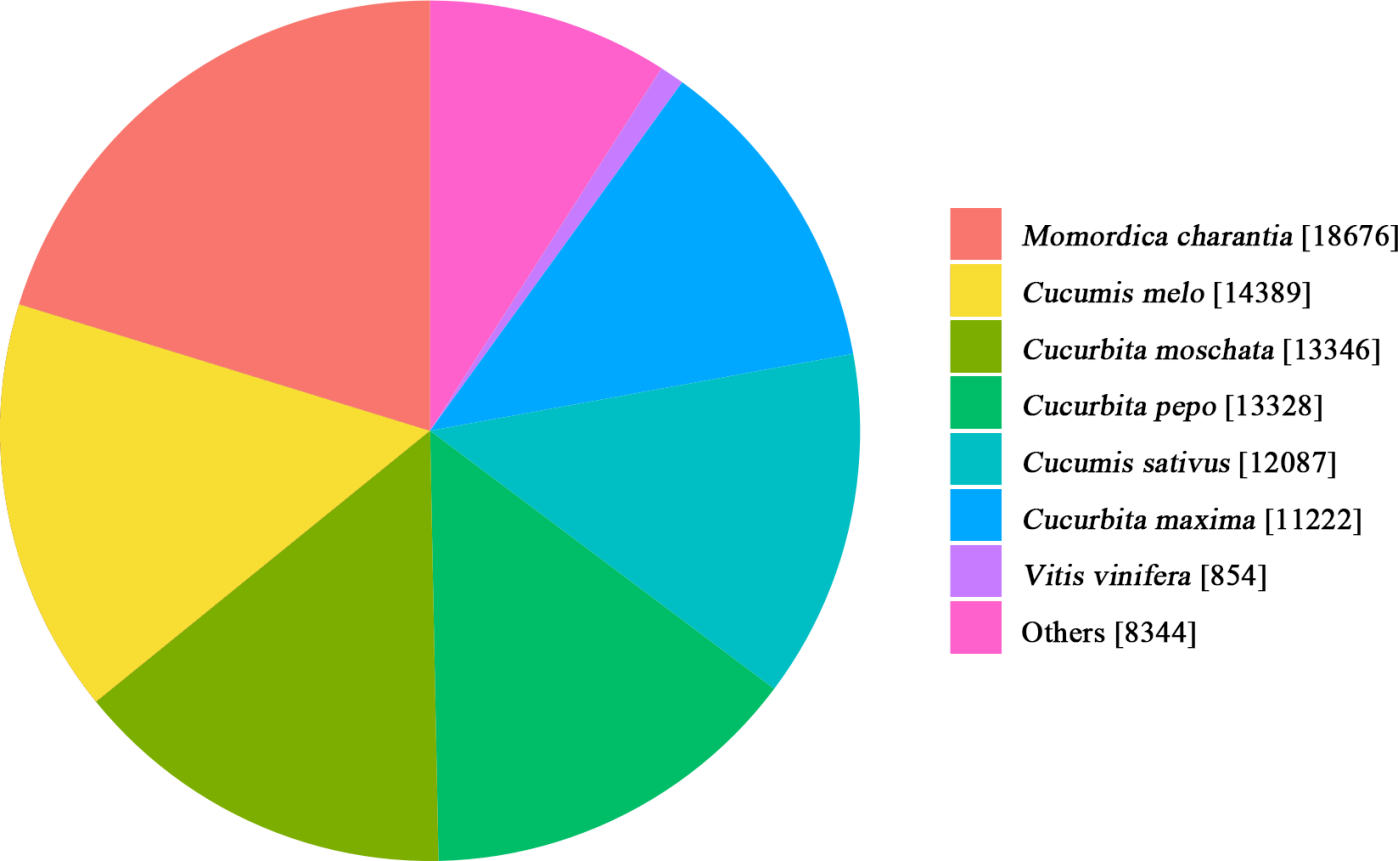
Species distribution of the *Luffa* unigene library against the NR database.

Comparison with the NR database showed similarity in the transcripts between *Luffa* and related species (Fig. 2). As shown in the pie chart, *Luffa* and *Momordica charantia* had the highest similarity, followed by *Cucumis melo*, *Cucurbita moschata*, *Cucurbita pepo*, *Cucumis sativus*, *Cucurbita maxima* and *Vitis vinifera* which have a great number of matches with *Luffa*. Except *Vitis vinifera* belongs to Vitaceae, the other species similar to *Luffa* all belong to Cucurbitaceae.

**Figure 3:**
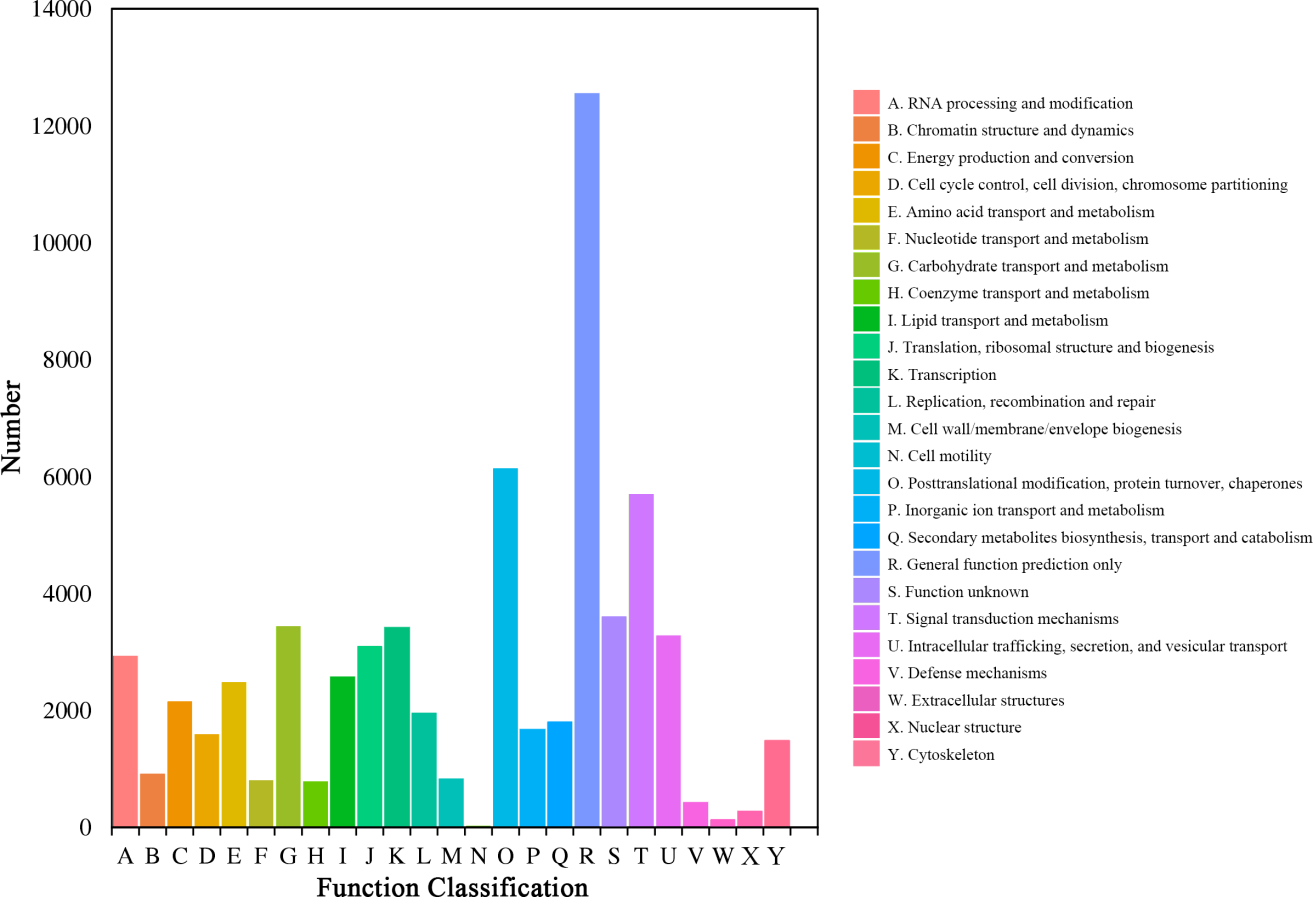
COG function classification of the *Luffa* unigene library.

The unigenes were also clustered by the ‘eukaryotic Orthologous Groups’ (KOG) of COG database. Based on the orthologous and evolutionary relationships, homologous genes from different species were divided into different clusters and the function of unigenes was annotated (Fig. 3). The top three categories among the all 25 categories were ‘general function prediction only’ (12,558, 19.59%), ‘posttranslational modification, protein turnover, chaperones’ (6,140, 9.58%), and ‘signal transduction mechanisms’ (5,702, 8.90%), respectively. However, ‘cell motility’ (24, 0.04%), ‘extracellular structures’ (132, 0.21%), and ‘nuclear structure’ (277, 0.43%) had the lowest percentage.

### Identification of DEGs

**Figure 4:**
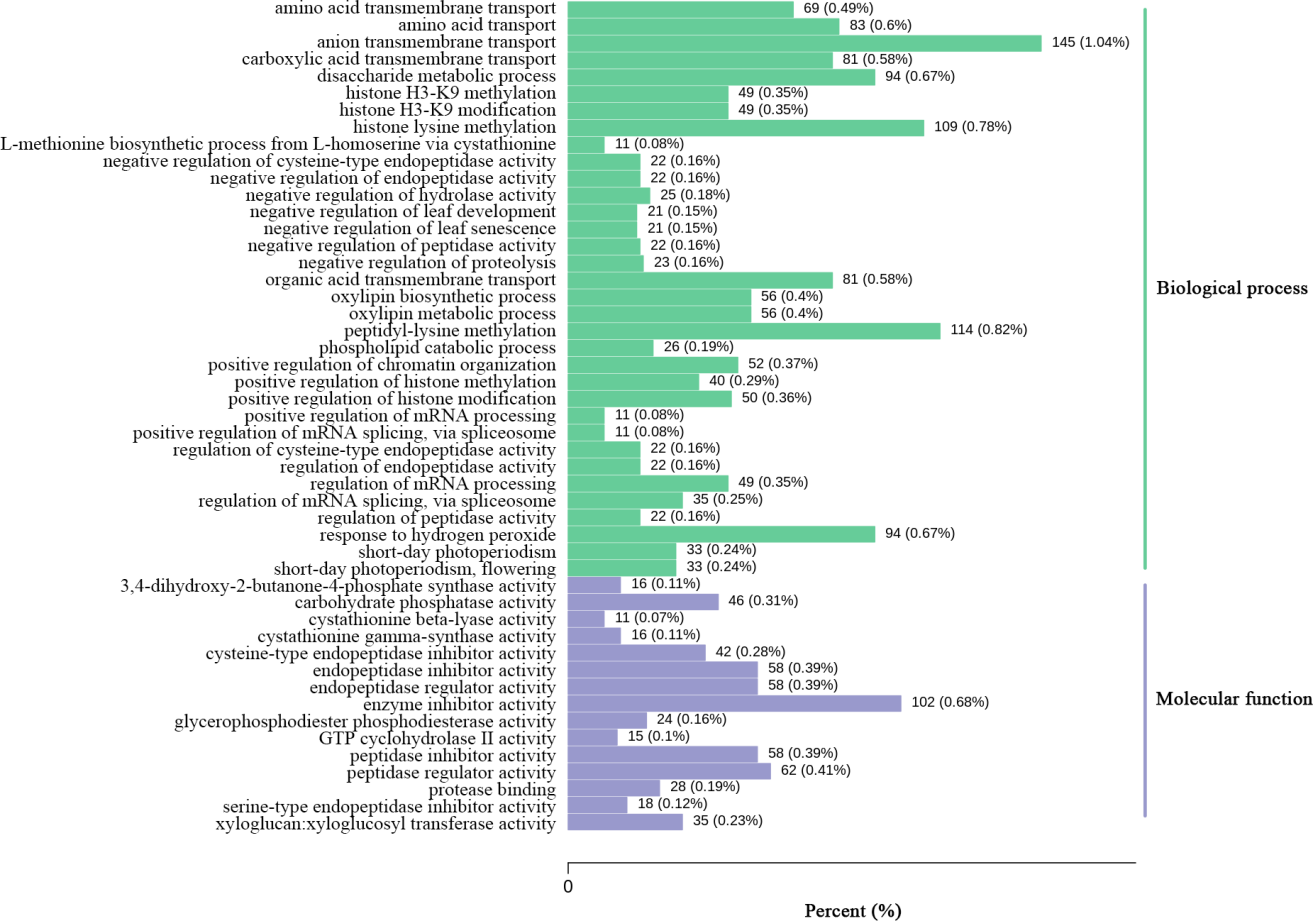
GO enrichment analysis of the DEGs identified in the unigene libraries between two *Luffa* cultivars.

**Figure 5:**
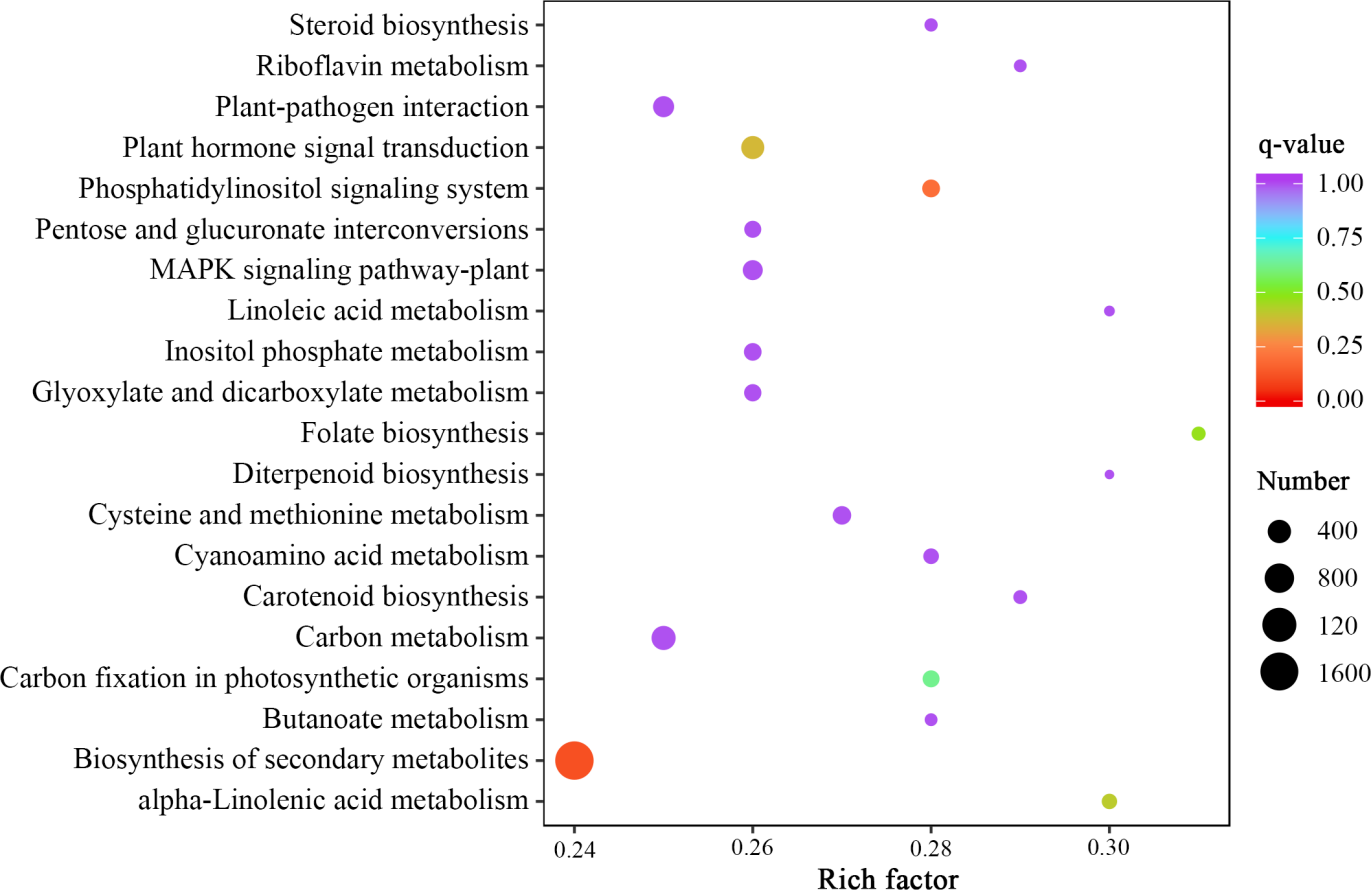
Statistics of KEGG enrichment of the DEGs identified in the unigene libraries between two *Luffa* cultivars.

By comparing the expression levels represented by FPKM of two *Luffa* cultivars ‘2D-2’ and ‘35D-7’, a total of 22,607 DEGs were identified. Of the DEGs, 13,465 were down-regulated and 9,142 were up-regulated in ‘35D-7’. The overall distribution of the expression levels and annotation of DEGs was determined (Table S2). The GO database was used to annotate the potential functions of the DEGs (Fig. S2). The top 50 GO-terms that possessed the lowest *q*-value are shown in Fig. 4. These GO-terms were distributed into two categories, ‘biological process’ and ‘molecular function’ based on background genes. The major GO-terms related to ‘biological process’ were ‘anion transmembrane transport’ (145, 1.04%), ‘peptidyl-lysine methylation’ (114, 0.82%), and ‘histone lysine methylation’ (109, 0.78%). As for the ‘molecular function’, ‘enzyme inhibitor activity’ (102, 0.68%) and ‘peptidase regulator activity’ (62, 0.41%) were the predominate GO-terms. The result of KEGG analysis (Fig. 5, Fig. S3) indicated that most of the DEGs were enriched in the ‘biosynthesis of secondary metabolites’ (ko01110) pathway (1,649, 21.47%). Pathways ‘plant-pathogen interaction’ (ko04626) and ‘carbon metabolism’ (ko01200) contained 291 (3.79%) and 444 (5.78%) DEGs. In ‘plant hormone signal transduction’ (ko04075), 386 (5.03%) DEGs were enriched.

### Identification of transcription factors among the DEGs

According to the rules of transcription factor family defined in plantTFDB database, several transcription factor families have been identified among the DEGs (Table 3). Twenty-six DEGs were classified into HB transcription factors and the number of up- and down regulated genes was similar. Meanwhile, 24 and 20 DEGs were identified as MYB transcription factors and C2H2 transcription factors, respectively, which were mostly downregulated. Other transcription factors, such as AP2, bHLH, bZIP, and NAC, were also annotated. Among the identified transcription factors, 47 DEGs only expressed in *Luffa* cultivar ‘35D-7’. The most significant difference in expression was found in Cluster-21832.27579 which was identified as bHLH transcription factor, followed by Cluster-10198.4 and Cluster-17143.0 belonging to Whirly and HSF family, respectively. On the contrary, a total of 107 DEGs were only detected in ‘2D-2’.

**Table 3:** Transcription factors identified in the DEGs.

**Transcription factor**	**Number of DEGs**		
	**Total**	**Up-regulated**	**Down-regulated**
AP2	16	9	7
B3	17	6	11
bHLH	14	8	6
bZIP	17	9	8
C2H2	20	5	15
C3H	11	3	8
FAR1	11	3	8
GARP	10	6	4
GRAS	7	5	2
GRF	4	2	2
HB	26	14	12
HD-ZIP	9	7	2
HSF	11	7	4
MADS	8	2	6
MYB	24	10	14
NAC	19	10	9
NF-YA	6	1	5
SBP	14	4	10
TCP	4	0	4
Trihelix	7	4	3
Whirly	2	1	1
WRKY	17	9	8

### Identification of browning-related DEGs in *Luffa*

**Figure 6:**
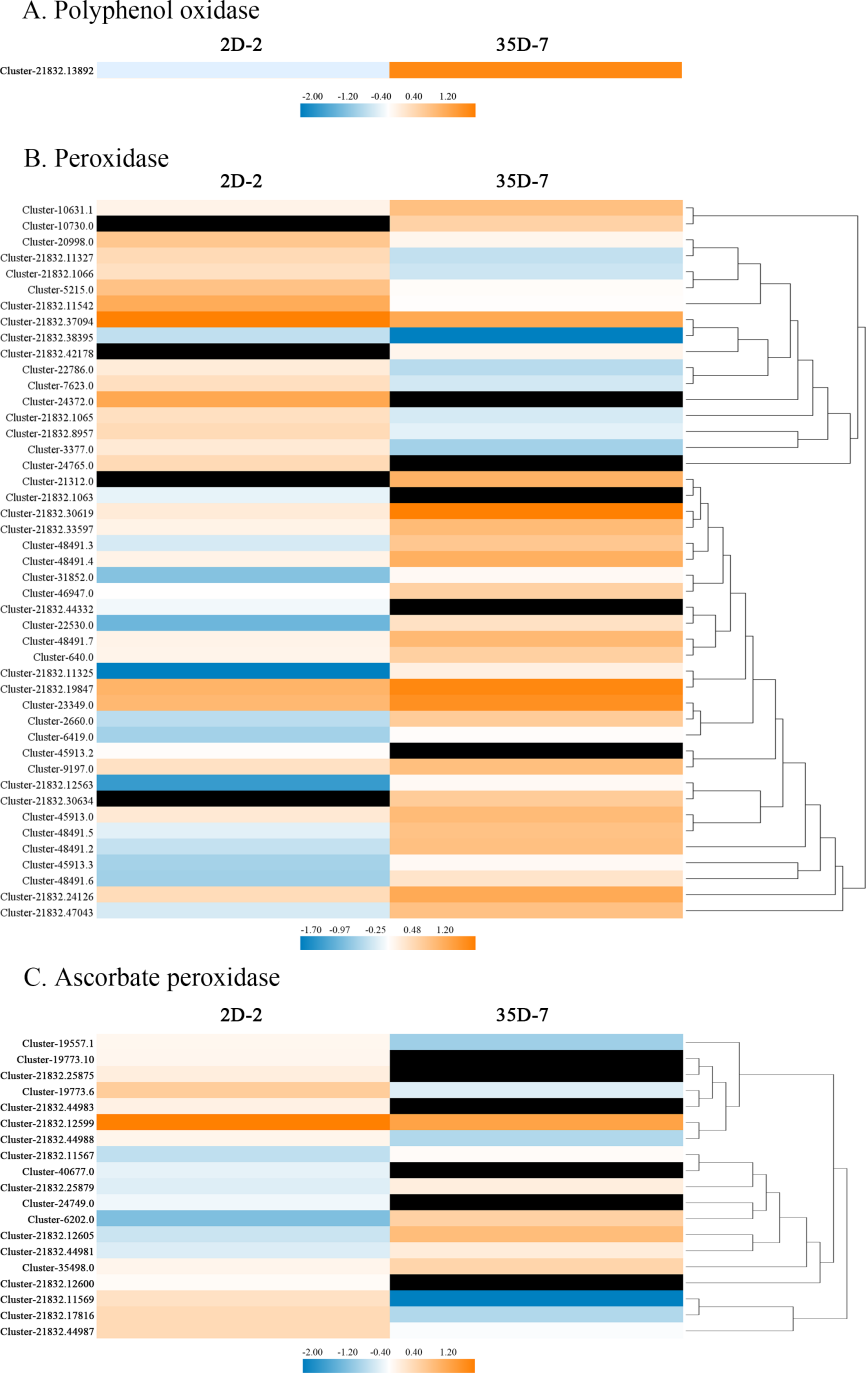
Heatmap of the expression levels of browning-related genes identified in the DEGs. (A) Polyphenol oxidase, (B) peroxidase, (C) ascorbate peroxidase. Heat maps were created by the log2 relative abundance of the DEGs. Blue and orange represent low and high expression, respectively. Black represents no expression detected.

To explore the gene regulatory mechanism of *Luffa* browning, we compared the expression levels of browning-related genes in the two *Luffa* cultivars with different resistance to browning. A total of 65 DEGs encoded PPO, POD, or APX were annotated to have roles in the enzymatic reaction in *Luffa* browning. Figure 6 shows the heatmap indicating the expression levels of these DEGs. The expression level of *LcPPO* (Accession No: Custer-21832.13892) was significantly higher in the *Luffa* cultivar ‘35D-7’ than in ‘2D-2’. Among the 45 DEGs related to *LcPOD*, 17 had lower expression and 28 had higher expression in ‘35D-7’ than in ‘2D-2’. In particular, the log2fold change of two *POD* genes (Accession No: Cluster-21312.0 and Cluster-21832.30634, respectively) reached up to 12.16 and 11.10, respectively. Nineteen APX-related genes were also identified, but most of them were down-regulated in ‘35D-7’.

### Validation of expression levels of browning-related genes in *Luffa*

**Figure 7:**
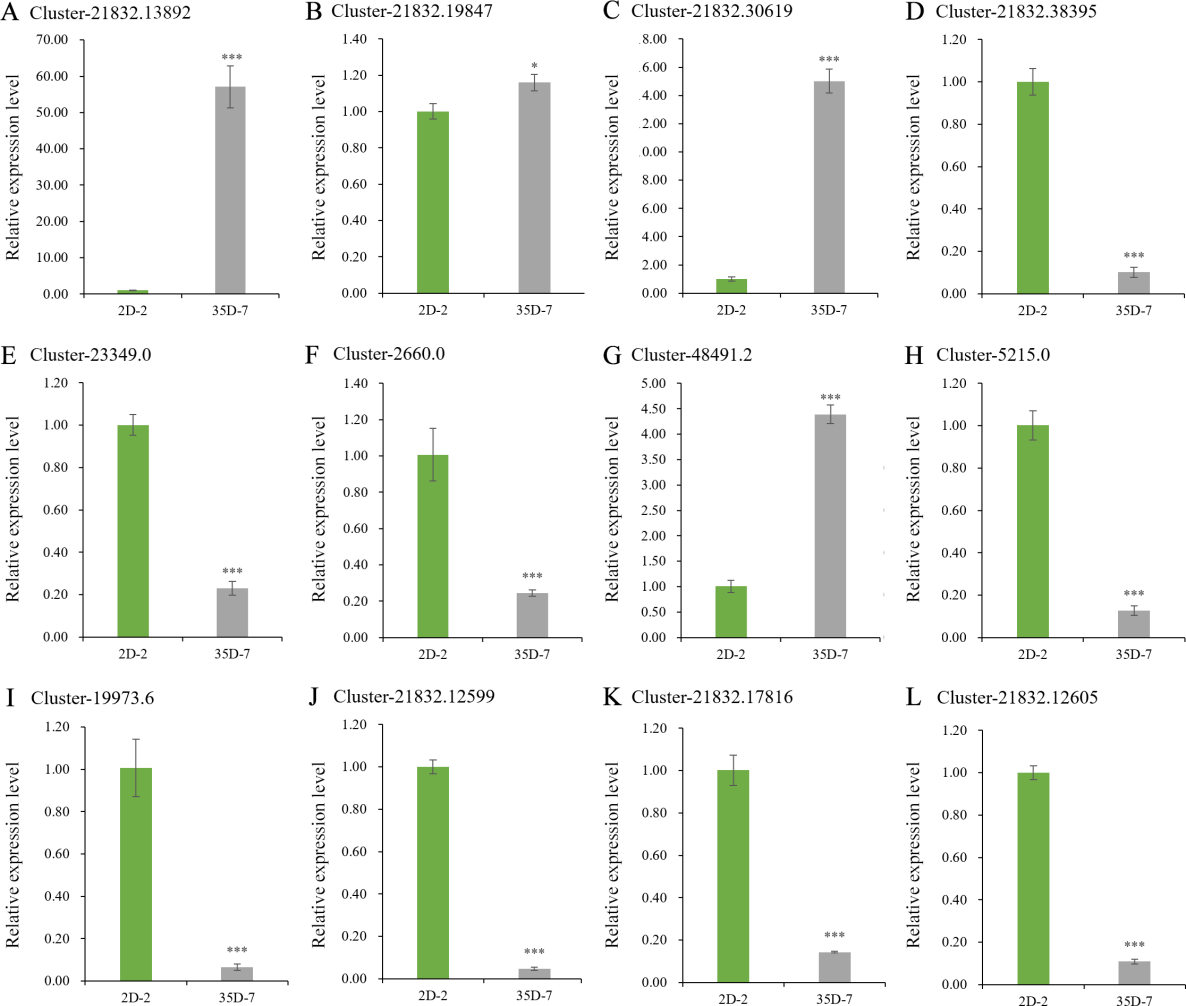
Expression levels of browning-related genes. The unigene ‘Cluster-21832.13892’ (A) was identified as *LcPPO* encoding polyphenol oxidase. The unigenes ‘Cluster-21832.19847’, ‘Cluster-21832.30619’, ‘Cluster-2183.38395’, ‘Cluster-23349.0’, ‘Cluster-2660.0’, ‘Cluster-48491.2’ and ‘Cluster-5215.0’ (B-H) were identified as *LcPODs* encoding peroxidase. The unigenes ‘Cluster-19973.6’, ‘Cluster-21832.12599’, ‘Cluster-21832.17816’ and ‘Cluster-21832.12605’ (I-L) were identified as *LcAPXs* encoding ascorbate peroxidase.

The expression levels of some DEGs, including *LcPPO*, seven *LcPOD* genes, and four *LcAPX* genes were detected and analyzed to validate the expression levels of DEGs related to *Luffa* browning (Fig. 7), their sequences are placed in the Data S1. The expression level of *LcPPO* (Accession No: Cluster-21832.13892) in ‘35D-7’ was 57.09 times that in ‘2D-2’. Among the selected *LcPOD* genes, three (Accession No: Cluster-21832.19847, Cluster-21832.30619 and Cluster-48491.2) were upregulated significantly in ‘35D-7’. The expression levels of all four *LcAPX* genes were lower in *Luffa* cultivar ‘35D-7’ than in ‘2D-2’. In general, nine of the 12 genes exhibited consistent expression trends in the results of RT-qPCR and transcriptome analysis.

## Discussion

*Luffa* is one of the most important melon vegetables, and its fruits are the main edible organ (Liu et al., 2017; Xu et al., 2018). Browning of fruits and vegetables is a complex process that involves several factors that work together (Ioannou & Ghoul, 2013). A number of methods, including physical, chemical, and biotechnology aspects have been studied to inhibit browning (Friedman, 1991; Chisari, Barbagallo & Spagna, 2007; Lopez-Nicolas et al., 2007; Arogundade & Mu, 2012). Studies on the browning tolerance of *Luffa* mainly focused on the physiological mechanism and variety selection. However, the related molecular genetic mechanism and genetic engineering have been rarely addressed. This lack of in-depth understanding affects the breeding progress of *Luffa* germplasm.

Transcriptome sequencing is one of the main methods used to explore gene expression, and is widely used in the fields of candidate gene discovery, functional identification and genetic improvement (Wang, Gertein & Snyder, 2009; Conesa et al., 2016). In the present study, we determined the transcriptome of two *Luffa* inbred cultivars ‘2D-2’ and ‘35D-7’. A total of 115,099 unigenes were clustered and annotated against seven databases. Among these unigenes, 22,607 DEGs with potential role in the regulation of *Luffa* browning resistance were identified. In 2017, Zhu et al. analyzed the transcriptome changes during browning and found that less unigenes (58,073) were assembled in the fresh-cut *Luffa cyindrica* ‘Fusi-3’ fruits (Zhu et al., 2017). These findings indicate differences in the transcriptomes of different varieties. The annotation against the KOG database was also similar to that reported in a previous study (Zhu et al., 2017). Comparison of the annotation of the unigenes shows that a large number of unigenes of *Luffa* were matched to *Momordica charantia* in addition to *Cucumis melo*. This result provides a new reference for the functional exploration of *Luffa* genes.

The identification of DEGs provides new insights about *Luffa* browning. Based on the results of the KEGG annotation, a large number of DEGs were enriched in the ‘biosynthesis of secondary metabolites’ and ‘carbon metabolism’ pathways. Phenols are the substrate of enzymatic browning. Phenols are important secondary metabolites formed by replacing the hydrogen atoms in the aromatic ring into hydroxyl or functional derivatives (Parr & Bolwell, 2000). The browning substrate of banana is dopamine (Griffiths, 1959). Browning in pear skin is caused by the phenols mainly composed of *l*-epicatechin (Siegelman, 1955). The main browning substrates of chufa are (-)-gallocatechin gallate, (-)-epicatechin gallate and (+)-catechin gallate (Sun et al., 2010). The polyphenol oxidase substrate content is likely different among different *Luffa* cultivars. DEGs related to plant hormone signal transduction and α-linolenic acid metabolism were also found in the two cultivars. Plant hormone is an important regulator of plant growth and development and participates in a variety of biological processes in plants (Friedman, 1991; Kulka, 2008). α-linolenic acid is a common fatty acid in plants that is mainly found in cell membrane lipids in plants (Li et al., 2016). The destruction of cellular structure occurs during browning. The different expression levels of α-linolenic acid-related genes demonstrated that cell membrane structure divergence may exist in different *Luffa* cultivars and may result in the easy destruction of the cell structure.

Among the identified DEGs, oxidase genes, which are thought to be involved in browning were the focus of this study. High expression levels of several genes were found in *Luffa* ‘35D-7’, including a *LcPPO* gene, a few *LcPOD* and *LcAPX* genes. RT-qPCR analysis was also performed to validate the expression of these gene in the *Luffa* samples. The *LcPPO* gene expression was extremely high in ‘35D-7’ and was more than 50 times that in ‘2D-2’. PPO is a metalloproteinase encoded by nuclear gene and can oxidize phenol substrates to quinone (Mayer, 2006). With the rapid development of cloning technology, *PPO* genes have been obtained from a variety of plants, including potato (Wang et al., 2003), wheat (Demeke & Morris, 2002), tea (Min, Liu & He, 2012), banana (Galeazzi, Sgarbieri & Conatantinides, 1981), etc. A close correlation exists between the expression levels of *PPO* genes and browning (Chi et al., 2014). In 2018, Zhu et al. cloned three *PPO* genes from *Luffa cylindrica* (L.) Roem and analyzed their expression profiles. The *PPO* genes expressed in different tissues of *Luffa* and responded to the postharvest storage and fresh cutting conditions (Zhu et al., 2018). In the present study, we found different expressions of *LcPPO* genes in different *Luffa* cultivars. This result indicated that *PPO* might be a key gene in regulating *Luffa* browning. Our results confirmed differences in the expression among different browning-resistant varieties. Several *LcPOD* genes were also found, but the expression differences were not as significant as *LcPPO* genes. In the presence of hydrogen peroxide, POD can catalyze the oxidation of phenols as a supplement to PPO (Chisari, Barbagallo & Spagna, 2007). Therefore, *LcPOD* genes were also involved in the browning process in *Luffa*. Previous study of Zhu et al. has confirmed that genes *PPO*, *PAL* (encoding phenylalanine ammonia lyase), *POD*, *CAT* (encoding catalase) and *SOD* (encoding superoxide dismutase) may associate with the enzymatic browning of fresh-cut *Luffa* (Zhu et al., 2017). We compared the expression levels of these genes in two *Luffa* varieties ‘2D-2’ and ‘35D-7’ and found that four genes, encoding PPO, PAL, POD and CAT enzyme, respectively, showed the same trend as the changes during browning of ‘Fusi-3’ (Table S3). The combined results demonstrated that some genes that play roles in the browning process also have effect on the browning tolerance of different *Luffa* cultivars. Further study on the functions of these genes will provide an important basis for the cultivation of *Luffa* varieties that are resistant to browning.

Transcription factors are proteins that can bind to specific sequences to regulate the expression of target genes (Katagiri & Chua, 1992; Schwechheimer, Zourelidou & Bevan, 1998). Transcription factors play an important role in plant growth and development, stress responses, and other aspects (Ramachandran, Hiratsuka & Chua, 1994; Meshi & Iwabuchi, 1995). We annotated the transcription factors according to the sequence obtained by the transcriptome analysis. A large number of transcription factors were identified among the DEGs, including some common transcription factor families AP2, MYB, WRKY etc. Our previous study found that some miRNAs related to browning can regulate downstream transcription factors (Xu et al., 2018). The result of Zhu considered that members of Group II WRKY superfamily may be related to the *Luffa* browning (Zhu et al., 2017). Furthermore, Liu et al. found that four *WRKY* genes were up-regulated in different postharvest and storage browning periods of *Luffa*, indicating their potential regulatory roles (Liu et al., 2017). In our study, 17 WRKY transcription factors have been identified among the DEGs. Based on the domain analysis, most of them had two WRKY domains which belong to the Group I subfamily (Table S4). The result suggested that more WRKY members may be involved in the regulation of browning. DEGs with high log2fold change including ‘Cluster-18244.0’, ‘Cluster-10926.1’ and ‘Cluster-18244.4’ need to be focused on. A large number of transcription factors were only expressed in one cultivar indicating their special roles. However, the functional identification of related transcription factor genes has not been carried out yet. Exploration of the functions of regulatory genes will be more helpful for *Luffa* breeding.

**Figure 8:**
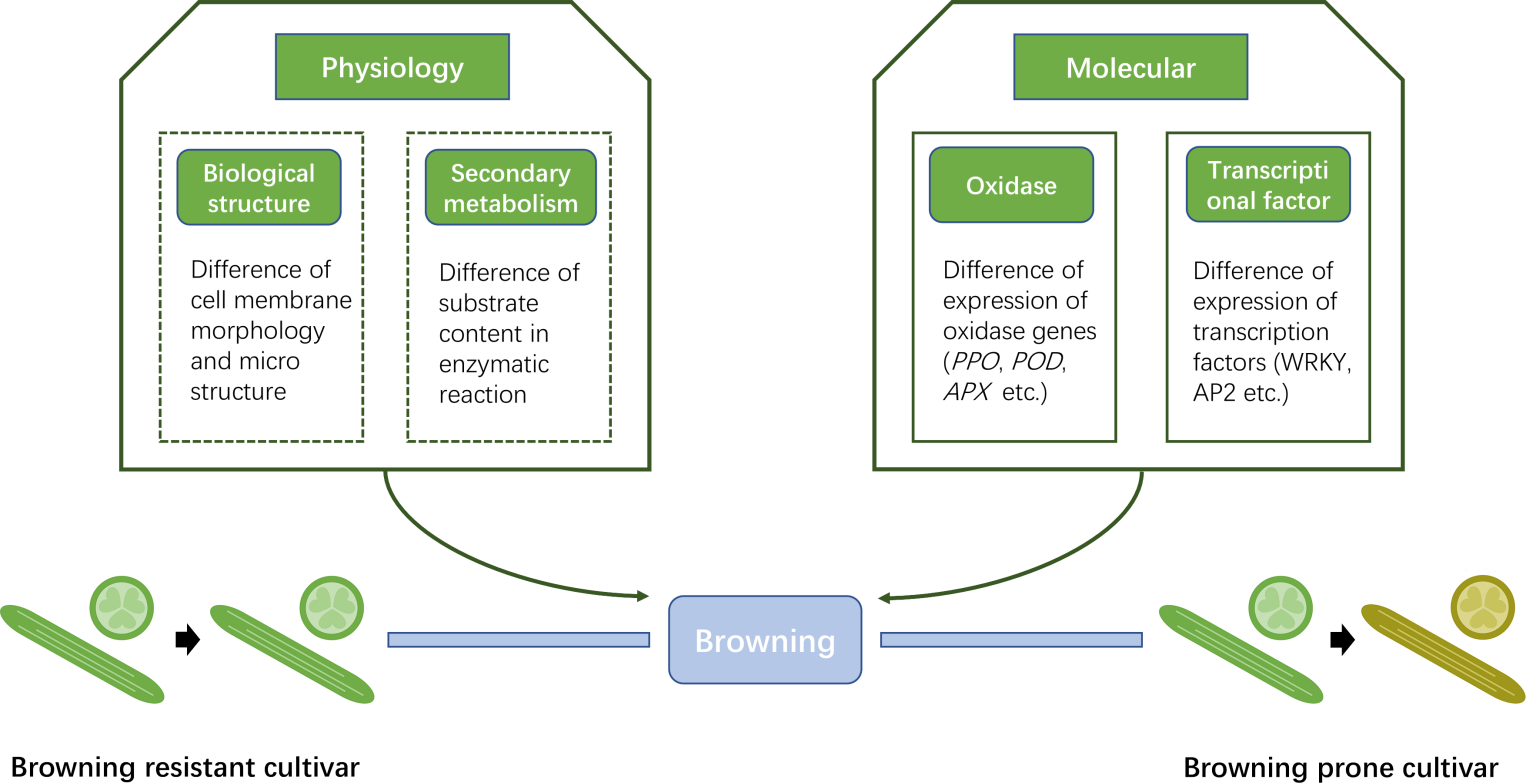
Simplified diagram of differences between browning resistant and browning prone *Luffa* cultivars.

We summarized a diagram indicating the new insights into browning-resistant *Luffa* cultivar (Fig. 8). From the viewpoint of physiology, the cell membrane structure varies among different browning-resistant *Luffa*. Divergence may also exist in the content of enzymatic reaction substrate that lead to browning. These two findings need to be further confirmed. In the present study, we confirmed the molecular differences between the two different *Luffa* varieties. The expression of oxidase genes, including *LcPPO*, *LcPOD* and *LcAPX* significantly differed between the two varieties. Multiple transcription factors such as WRKY transcription factor family may also play regulatory roles. Our findings provide potential insights into the molecular regulation of *Luffa* browning and establish foundation to cultivate improved *Luffa* germplasm with high browning resistance.

## Conclusions

Our study compared the transcriptomes of two *Luffa cylindrica* cultivars with different resistances to browning by high-throughput sequencing. Among the differentially expression genes, 65 encoded polyphenol oxidase (PPO), peroxidase (POD) and ascorbate peroxidase (APX) were identified and further analyzed. The result of RT-qPCR confirmed the expression levels of 12 selected genes and demonstrated that these genes expressed higher in *Luffa* cultivar with strong browning resistance. Some transcription factors such as WRKY gene family may also participate in the regulation of *Luffa* browning. Our findings can establish foundation to cultivate improved *Luffa* germplasm with high browning resistance.

##  Supplemental Information

10.7717/peerj.9661/supp-1 Supplemental Information 1 Annotation of the unigenes based on seven databases

10.7717/peerj.9661/supp-2 Supplemental Information 2 DEGs identified between two *Luffa* cultivars

10.7717/peerj.9661/supp-3 Supplemental Information 3 Supplementary figures and tables Figure S1: GO classification of the *Luffa* unigene library, Figure S2: GO classification of the DEGs identified in the unigene libraries between two *Luffa* cultivars, Figure S3: KEGG classification of the DEGs identified in the unigene libraries between two *Luffa* cultivars. Table S3. Comparison of unigenes identified in transcriptome during browning of ‘Fusi-3’ fruits and transcriptomes of two *Luffa* cultivars ‘2D-2’ and ‘35D-7’. Table S4. Information of WRKY transcription factors identified in the transcriptomes of *Luffa* ‘2D-2’ and ‘35D-7’.

10.7717/peerj.9661/supp-4 Supplemental Information 4 Sequence of the selected DEGs for gene expression analysis

10.7717/peerj.9661/supp-5 Supplemental Information 5 Raw data for qRT-PCR: Cycle threshold values of candidate genes in RT-qPCR analysis

## Additional Information and Declarations

### Data Availability

The following information was supplied regarding data availability: The raw data for qRT-PCR are available in the Supplementary File. The sequencing raw data are accessible in the Sequence Read Archive (SRA) database: SRP252794. Assembly sequences are available at TSA database: GILZ00000000.
