# Supplementary material for: Sequencing, assembly, annotation, and gene expression: novel insights into browning-resistant Luffa cylindrica
Source: PeerJ. 2020 Aug 10;8:e9661. doi: 10.7717/peerj.9661 (PMC7425639; doi:10.7717/peerj.9661)
Supplement: Supplemental Information 3 — Figure S1: GO classification of the Luffa unigene library, Figure S2: GO classification of the DEGs identified in the unigene libraries between two Luffa cultivars, Figure S3: KEGG classification of the DEGs identified in the unigene libraries between two Luffa cultivars. Table S3. Comparison of unigenes identified in transcriptome during browning of ‘Fusi-3’ fruits and transcriptomes of two Luffa cultivars ‘2D-2’ and ‘35D-7’. Table S4. Information of WRKY transcription factors identified in the transcriptomes of Luffa ‘2D-2’ and ‘35D-7’. [file peerj-08-9661-s003.pdf]

Supplemental Information S1:

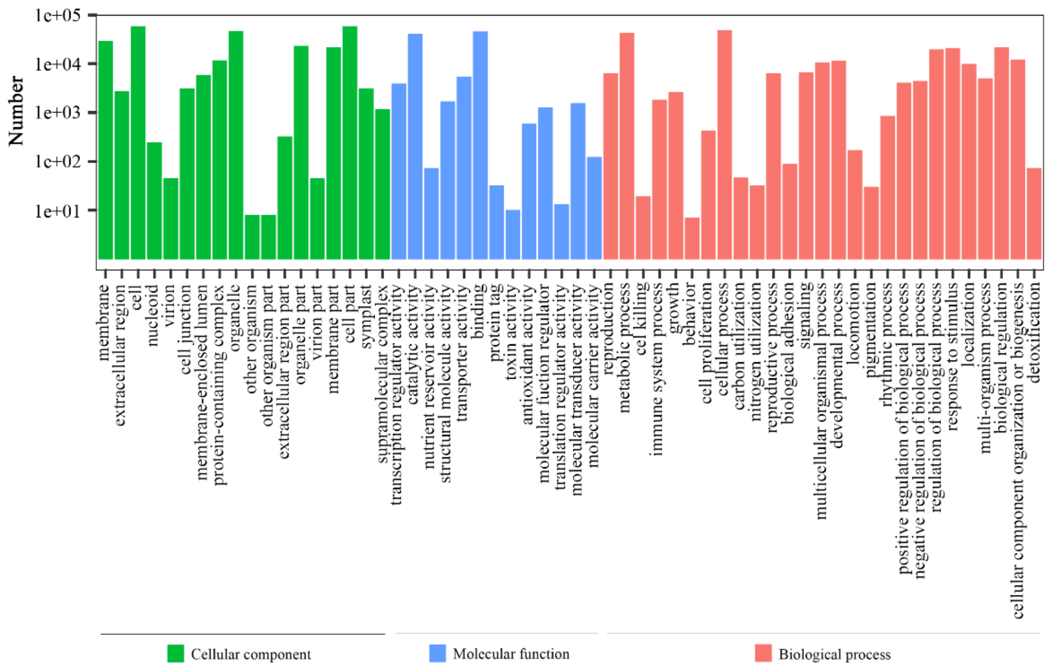

Figure S1 GO classification of the *Luffa* unigene library

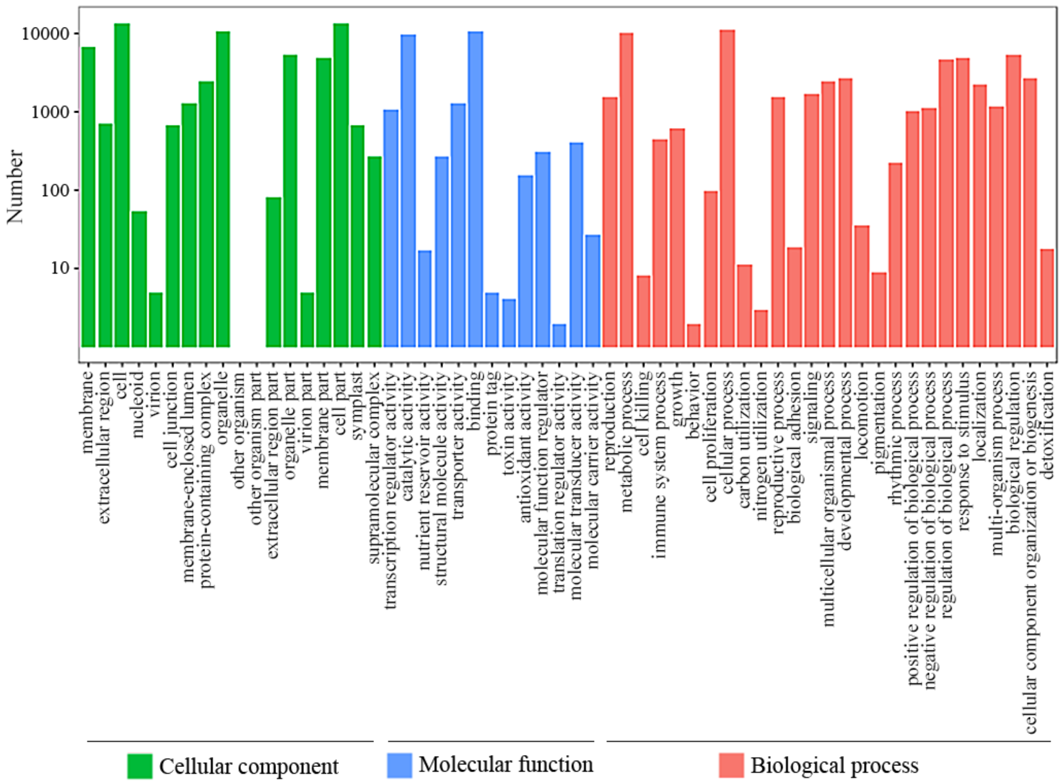

FigureS2 GO classification of the DEGs identified in the unigene libraries between two *Luffa* cultivars.

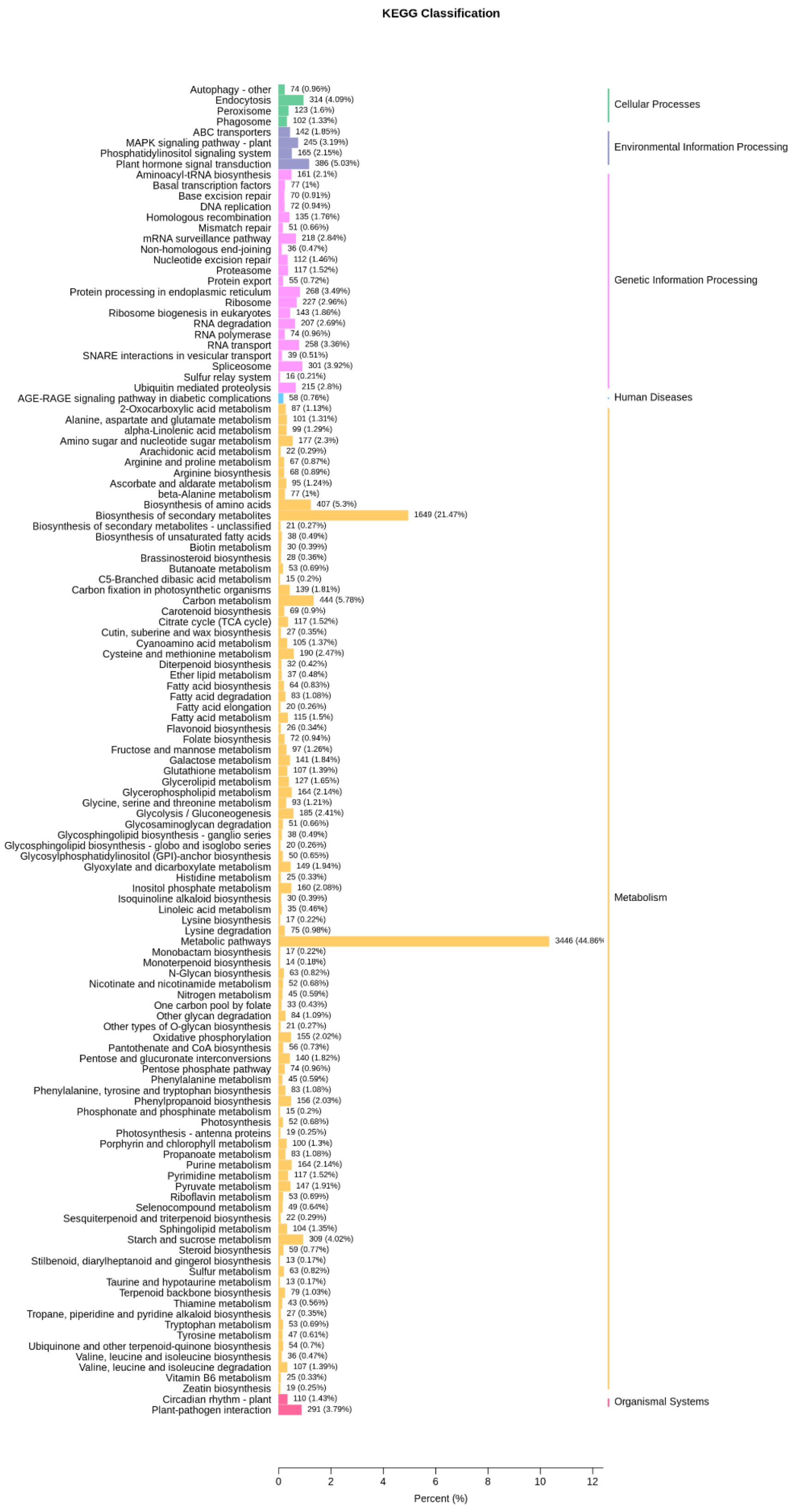

Figure S3 KEGG classification of the DEGs identified in the unigene libraries between two *Luffa* cultivars.

Table S3 Comparison of unigenes identified in transcriptome during browning of 'Fusi-3' fruits and transcriptome of two *Luffa* cultivars '2D-2' and '35D-7'.

**'Fusi-3'**		**'2D-2' and '35D-3'**			**Annotation**
**Unigene ID**	**Expression trend**	**Unigene ID**	**Expression trend**	**Log2fold change**	
Unigene 0043875	up	Cluster-21832.13892	up	7.15	polyphenol oxidase
Unigene0050171	up	none	none	none	polyphenol oxidase
Unigene0015096	down	Cluster-21832.13892	up	7.15	polyphenol oxidase
Unigene0044748	up	Cluster-21832.39888	-	-	phenylalanine ammonia-lyase
Unigene0024978	up	Cluster-47379.0	up	2.16	phenylalanine ammonia-lyase
Unigene0011770	up	Cluster-21832.19847	up	2.27	peroxidase
Unigene 0036262	up	Cluster-21832.36955	up	2.72	catalase
Unigene0033876	down	Cluster-21832.30636	up	2.21	catalase
Unigene 0021782	up	Cluster-21832.29146	-	-	superoxide
Unigene0008835	down	Cluster-45332.0	-	-	superoxide
Unigene0015506	down	Cluster-21832.7745	up	9.46	superoxide

'Fusi-3' represents the transcript analysis during the browning of 'Fusi-3' fruits. '2D-2' and '35D-7' represents the transcript analysis of browning-resistant *Luffa* cultivar '2D-2' and browning-prone *Luffa* cultivar '35D-7'. 'None' indicates no matching to the sequence. '-' indicates that no significant difference was found in gene expression. Green and red color represents the same and opposite expression trend, respectively.

Table S4 Information of WRKY transcription factors identified in the transcriptomes of *Luffa* '2D-2' and '35D-7'.

Unigene No.	Expression log2FoldChange	Regulated	Domain Number	Description	Accession No.
Cluster-18244.0	12.4292	up	2	WRKY DNA-binding domain	pfam03106
Cluster-10926.1	10.0614	up	2		pfam03106
Cluster-21832.2248	3.58428	up	1		smart00774
Cluster-21832.38074	2.78687	up	1		smart00774
Cluster-21832.39105	2.34574	up	2		pfam03106
Cluster-21832.6008	2.27242	up	2		pfam03106, smart00774
Cluster-21832.26783	2.06106	up	1		smart 00774
Cluster-18244.1	1.99927	up	1		pfam03106
Cluster-10926.2	1.79678	up	2		pfam03106
Cluster-21832.19413	-1.7055	down	2		pfam03106
Cluster-21832.40922	-2.4811	down	2		pfam03106
Cluster-21832.22382	-2.7626	down	2		pfam03106
Cluster-21832.19423	-9.7218	down	2		pfam03106
Cluster-21832.14119	-9.8482	down	2		pfam03106
Cluster-21832.18531	-9.8809	down	2		pfam03106
Cluster-21832.40775	-9.9233	down	2		pfam03106
Cluster-18244.4	-12.376	down	2		pfam03106
