# Supplementary material for: Sequencing, assembly, annotation, and gene expression: novel insights into browning-resistant Luffa cylindrica
Source: PeerJ. 2020 Aug 10;8:e9661. doi: 10.7717/peerj.9661 (PMC7425639; doi:10.7717/peerj.9661)
Supplement: Supplemental Information 4 [file peerj-08-9661-s004.pdf]

Data S1: Sequence of the selected DEGs for gene expression analysis

Cluster-21832.13892

>TATTTCGTCCCACTTTTCCGTAACAACAAATACCCATCATTCGGCCCTCGAAGCAGAA
AGTTTCTCATCAACAAAGCTCCTCGAGTTTTGTGCAGCGCCTCCAATGGCGACCAAAA
GTTCGACCGGAGAGACGTGCTCCTTGGGCTGGGCGGTCTCTACGGAGCCTCAACTCTC
GGCCCGGATTCCTTCGCCTTCGCCAGTCCGGTGGTGACGCCGCCACTGGACAAGTGTC
ACTTAGCAACCTTGCCAGATGGCACAAAGGATGCTTGCTGCCCGCCGACGGCCGTCAA
CATCAAAGACTTCCGGCCAACCCCACCTAGCAAACTGCGGGTTAGGCCGGCGGCCCAT
TTAATTGATGAAAAGTACATAGAGAAATACAAGAGAGCCGTCCAGCTGATGAAGTGCT
TGCCGGACAACGACCCAAGAAATTTAGACAGCAAGCGAATGTTCACTGTGCCTATTG
TCATGGCGGCTACGACCAAGTGGGGTTTCCAGTGCAGGTCCAAGTTCATAACTCGTGG
CTGTTTTTCCCATTCCATCGTTACTATCTCTACTTTTACGAGAAGATCTTGGGAGAGCTG
ATCGGTGATCCCACTTTCGGGTTGCCGTTTTGGAACTACGACGCTCCGAAGGGCATGA
GAATGCCGGCCATCTTCGCCGACAAGAACTCGCCGCTTTACGATCAAGTTCGTAATCTA
AATCATCTGCCGCCGGCGTTAGTGGATCTGAATCTGGATGACGCGGAAACAGACGAAG
AAACTCTAGTAAAGAGAAACCTCCGGGTTATGTACCGGCAGGTGGTGTCCGGCGCCCG
TACGCCGTCTCTGTTTTTTGGCGTCCCTTATCGAGGGGGTGATGCAGCACCAGAACAA
GGAGGAGGATCTGTGGAAAACGTTCCTCACACCCCGATCCATATTTGGTGCGGCGATA
GCGACCAAGGAGGTAGGGACATGGGAGCTTTCTACTCCGCCGCTCGTGACCCAATCTT
CTACGCCCACCACTCCAACGTCGACCGCCTTTGGTCCATCTGGAAGACCCTCGGCGGC
AAGCGCGAAGACCTCAAAGACCCTGATTATCTCAACTCTTCTTTCATATTCTACAACGA
GAAGGCCGAAGCCGTCCGAGTCTACGTCCGCGACTGTCTCGACCTCAAAAACCTCGG
CTACGTCTACCAGGACGTCGACATTCCATGGATCAAGACCCGCCCTACCAAACGCAAC
AAGCCCAAGAAGAAGCCCAAGAAACAAGTGGCCTCCACCGTCCCCTTCGGCGTCGGC
GCAGCCCTCGCCGAGCCGATCTCGTTCACCGTCGAGAAGGCGGGCATGGCCCAGTTCC
CCTTGACTCTGGACAAGATCGTCAGGACGGAAGTGAAGAGGCCGAAGAAGTCGAGG
AGCAAGGAGGAGAAAGAGGAGGAAGAGGAGATTCTGCTGATCGAAGGAATCGAAGT
CGACGCCGGCAAGTCGTCAAAGTTCGACGTCTATATCAACGACGAGGACGACAGGGA
AATTGGACCTGATAATACTGAGTTTGCTGGGAGCTTTGTGAATTTGCCTCATAAGGTGA
GCGGCAAAAATACGACGACCTTGAAGACCTCCCTTAGGCTGGGGCTCAACGAACTGC
TTGAAGATTTGGAGGTGGATGATGATGAAAGCATCGTGGTCACTCTGGTGCCCAAGTT
TGGATTCGACCAATCGCCC

Cluster-21832.19847

>ATGGCATCTTTCAATGTTGCAGCCTTCCTTTTCTTTCTGGGTCTTTTGTTTGGAGCCTC
TTGCCCAACTCACCGAGGACTTCTACGACCAAACTTGCCCTCGTCTCCCCAACATTGT
CCGCCGTGAGGTCAAGAGAGCTATCGAATCCGACATCCGTGCCGGGGCCAAACTCAT
CCGCCTCCATTTTCATGACTGCTTCGTCCAAGGCTGCGATGGCTCTGTTTTGCTAGTGG
ACGCTCCTGGCATAGAGAGTGAGCTCAACGGACTTGGAAATTTAGGAATCCAAGGAC
TCGAAATTGTCGCCGCCATCAAAGCCGCCGTCGAGAGGGAATGTCCCGGCGTCGTCT
CCTGCGCCGACATCCTAGCTCAGGCCTCTAAAGACTCTGTCGACGTGCAAGGAGGGC
CTAGTTGGAGAGTTTTATACGGAAGAAGAGACAGCAGAACAGCCAATAAAACAGGG
GCTGATGAACTCCCAAGTCCCTTCGAAACTCTCGAGCCACTCAAGAAAAAGTTCGAA

GCGCTTGGCCTCAATTCCACCGATCTGGTCGCTCTATCTGGGGCACATACGTTCGGTC
GGTCGAGATGCAGGTTCTTCAACGGGCGTTTCTCGAACTTCAGCGGAAGTGGGCAGC
CAGATCCGACGCTGGACCCAACGTACAGGCAAGAGCTGGAAAGAGCTTGTACAAATG
AAGACACACGAGTGAATTTCGATCCAACAACACCGGACACATTCGACAAGAATTACT
ACACGAACCTTCAAGCCAATCGAGGGCTTCTAACGAGCGACCAATCGCTGTTCTCCG
AGGCAGGGGCGGACACAGTCGACACTGTCAACCTTCTTGCGTCGCGAGAAGGCACCT
TCTTAAGATTCTTTAGGCGATCCATGATTAAGATGGGCAATATCAGACCTTTAACTGG
AAACCAAGGGGAAATCAGAACAAACTGCAGCAGGCTCAATGACTTGGGAGCTCAAC
CAGGCCACGATGTTATGTT

Cluster-21832.30619

>ATGGCTTCCCCTAAACTTACAGCCTTTGCCCTGGTGCTGGCTTTGTTGCTGGCGACCT
CTCAAGCTCAGCTTAACCCTTTCTTCTACGCCTTAACATGCCCTCTCCTGCCCGGGATT
GTTCTCGACGTCGTCTCCCGAGCTCTACAAACCGACGATCGAGCAGCTGCCAAGCTCA
TTCGCCTCCATTTCCACGACTGCTTCGTCAATGGATGTGACGGTTCTGTGCTGCTGGAA
CCCGCACCGGGCATCGACAGTGAATTAAACGGGCCACCAAACAATGGGATTCAAGGG
CTAGACATAGTAGACAACATCAAAGCAGAACTTGAAAGATTTTGCCCCGGCGTCGTTT
CATGCGCTGACATCTTAGCCATTTCCTCTCAAATTTCTGTTTTCTTGTCGGGAGGGCCA
GCTTGGATGGTTCCATTGGGAAGAAAAGACAGCAGAATAGCCAACAGAACCGGAACC
TCAAATCTTCCTGGTCCATCAGAACCTCTTACCGGACTTAAAGCCAAGTTTGGAGCTCT
TGGGTTCGATTCTACGGATCTGGTGGCTCTATCAGGAGCACACACGTTCGGTAAAGCA
AGATGCCTTTTCTTCAACGGCCGCTTCGACAACTTCAACAACACCGGCAAACCCGACC
CGACGCTAGACCCGACTTACAGAGAGCAGCTTCGGAGACTATGTACGACCCCACAAAC
ACGAGTGAATTTCGACCCGACCACGCCAACAAAATTCGACAAAGCCTACTATTCCAAT
CTGCTGAGCCTCAAGGGGCTTCTCCAAAGTGACCAAGAGCTGTTCTCCACGCCCAGG
GCTGATACCACGGCGATCGTAAAGAATTTCGCCGCTAACGAGATTGCGTTCTTTAATCA
ATTTGTGAAATCGATTATCAAAATGGGGAATCTCAAAGCTCCGGCCGGCATCAGATCAG
AAGTGAGATTAAACTGTAGGAGGGTCAACCCGGTGGCTGCCCATGATGTTATG

Cluster-21832.38395

>ATGGATGGGAGGGAAGGCATGGCATTGTCTGGTGGCTCTGCTTCTTATTACATACAT
AGGGGAGGAGGGGTTGGTGGCTCTGGATCAGGGCTGCCTACGGCTGGATCACATGCC
TCCCCTGTATTCCGGCCAATGCCTAACCAGGGTGTTTTGTCTCACTCAAATCTCAGAG
GCAACTCAGTTGGATCAACATATGCTGTTGAGCCCCCACATCCAAATTATCTTCGTGG
GATGAGTATGAATGTCTCAGCTGGAGTGAACTCTGGTGAGCCTGTAAAGAAGAAGAG
AGGGAGACCAAGGAAATATGCTCCAGATGGGCAGGTCTCATTGGGTCTGTCTCCCAT
GTCTGCCGGTTCTAAGCTCACTCCAGGTTCGAATTCGTCGACCCCGAGACGTCGAAGA
GGGCGGCCACCTGGTTCTGGAAGGAAACAACAATTAGCCCTTCTTGGTGAGTGGATG
AACAATTCAGCTGGACTAGCTTTTGCCCCTCATGTCATTCACGTTGGAGCTGGAGAAG
ACATTGTGGCAAAAGTATTGTCGTTTGCACAGCAGAGGCCAAGGGCTGTTTGCGTCCT
GTCAGGCAACGGTACTGTTTCTTCAGTAACACTTCGTCAGCCTGCATCTACTGGTGTG
AGCGTCACATATGAGGGTCATTTCCAGATATTGTGCTTATCTGGTTCTTACTTGGTGGC
TGAAGATGGTGGTCCTCGAAATAGGATGGGCGGTATTAGTGTTTCCCTCTCTAGTCCT
GATGGTCATGTCATTGGTGGCGGTGTTGCCGTTCTTATGGCAGCTGGCCCCGTTCAGG

TACTT

Cluster-23349.0
>TTGCAGGATATTCGTGGAAATGATGTGAGTCTTAGTCAATACAGGGGGAAGGTTCTTC
TGATAGTGAATGTTGCTTCTCAGTGTGGTTTAACGCAGTCAAACTACAAGGAGTTGAAT
GTATTGTATGAAAAATACAAAAATCAAGGTTTTGAGATATTGGCATTTCCTTGCAATCAG
TTTGCATCACAAGAGCCAGGAAGCAATGAGGAGATTCAGGAAACTGTATGCACAAGG
TTCAAAGCTGAATTCCCCATCTTCGATAAGGTTGAGGTTAACGGGAAGAATGCAGCAC
CCATTTATAGGTTCCTGAAATCACAAAAGGGTGGAATATTTGGCGATGGCATCAAATGG
AACTTCACAAAGTTCTTGGTGAACAAAGAAGGGAAGGTGGTTGATAGATATGCTCCGA
CCACCTCGCCTTTCAAGATTGAGAAAGATATCCAGAGTCTATTGCAATCTGCT

Cluster-2660.0
>GGCTGTGATGGATCATTGTTGCTTGATGACACTAGCACATTTAAGGGGGAGAAAAA
TGCATTGCCGAATATGAACTCAGTTCGAGGCTATGAGGTCATTGATAATATAAAGGCT
GTTTTAGAGAGAGCCTGCCCATCCGTCGTCTCATGTACCGATATAGTAACTCTAACAG
CTAGAGAGGCCGTCTACCTTGCTGGAGGACCATTTTGGCCCATTCCACTTGGTCGCCG
AGATGGCACGACAGCGAGCGAGAGTGAAGCCAATCAGCTGCCATCACCAGTGGAGC
CATTGGAGAATATCGTTGCAAAGTTCACTTCAAAAGGTCTTGATGTAAAGGATGTTGT
TGTGCTCTCAGGTGCACACACTTTTGGATTTGCTCGGTGCATGATGTTCAAGCATAGG
CTCTTCAACTTCGACGGCTCTGGAAATCCCGATCCAGAACTCGACGTGATGCTACGAC
AGAACTTACAGAGCACCTGCCCAAATCAAGATGACTCCAACAACAACCTGGCTCCCT
TGGACGCCTACACGACCAACAGGTTCGACAATGTGTATTACAGGAACTTGGTGAACA
AGTTGGGACTTCTGCAGTCAGACCAAGCCCTCATGAATGACAATACAACTGCTCCTTT
GGTGGTGAACTACAGCAGGTACCCATATCTGTTTTACAGGGACTTTGGAGCATCAATG
GTGAAGCTGGCCAATACTGGGATCCTTACAGGCCAAAATGGCGAGATTAGGAAGAAC
TGCAGGGTCGTGAAC

Cluster-48491.2
>TTACCAAACACTCATACTTTGCTTGCCGGACTCCGTGCCAATTGGTATGGAATTTTG
GGAAAGAAATTCAAGGACACATTTGGACACATCGGGGGATCCATACTCGGAGGTTTA
GTGGGCTTGAAGAAACCTAATAATCACAACATCCCTTATTCCATGACCGAAGAATTTA
CGAGTGTTTATCGAATGCACTCCCTCTTACCTGATGATTTTCATTTAAGAGATGTTTCC
ATTGATCCAGATCACAATAAATCTCCACCATTAATTCACAAAGTGCCAATGGTAAAC
ATGGTGGGCCTTAAGGGAGAGCAAGCATCAAAGGAAATGGGCTTCACGGCCCTGATC
GTTTCCATGGGCCACCAAGCCAGTGGAGGCCTAGAGCTTTGGAATTATCCGCTGTGGC
TGAGGAATCTTACAGCTCATGACGTGGACGGCAAAGATAGGCCCGACCACGTTGACC
TGGGTGCCCTCGATGTTTACAGAGATAGGGAGAGGAATGTTGGTAGGTATAATGGCT
TCCGTAGGGGATTGCTGCTGAAACCAATCTCCAGATGGGAAGATCGGACGGATGACA
ATGAGGCGATTGAAGCTCTCGGTGAAGTGTACGGTGATGATGTGGAGGAGCTAGCTT
GATATTCTTGTAGGTCTGATGGCAGAGAAGAAAATGAAGGGCTTCGCCATTAGTGAG
ACTGGTTTTATGATTTTCTTGTTGATGGCTTCCAGGAGGATAGAGGCAGATAGATTCT
TCACAAGCCATTTCAATGAAGAGACATACACAAAGAAGGGATTGGAATGGGTGAAG
AGTACAGAGAGGTTGAAGGATGTGATCCTGACATCTCAAACAAATGGATAAACTCAT

CAACTGCTTTCTCAGTTTGGGACTCTCCTCCCAACAAACCCAACCCTATCCCAATTTA
CCTTCGAATTCCA

Cluster-5215.0

>CGGAGCTTGTATGAGACAGGTGACCCCCCATTGACGTTGGATTACTATGCCAAAACT
TGTCCCAATGTGTTGCAGGTTGTGAGGAAAGAGATGGAGTGTGCAGTGCTTTCTGACC
CACGTAATGCAGCTTTTGTTGTCCGATTGCACTTCCACGACTGCTTTGTCCAGGGGTG
CGACGGGTCGGTTCTGCTGGACGACACTATAACATTACAGGGAGAAAAAAAGGCTTC
CACCAACATACACTCCCTCAAAGGCTTCAGAATCATAGACAGGATAAAGAACAGCCT
TGAATCAGAATGTCCTGGGATCGTTTCTTGTGCAGATATACTCACTGTTGCAGCCAGG
GATGCAGTGATTCTGGTAGGCGGCCCTTACTGGGAGGTTCCTCTTGGAAGAAAGGAT
TCCACAACTGCGAGTTATGAACTTGCAAGCACAAATCTTCCCTCTGCCAACGAGGGG
CTTCTGAGCATCATTTCCAAGTTTCTTTATCAGGGTCTCTCTGTCACTGACATGGTAGC
TCTATCAGGAGCGCACACTATTGGAATGGCAAGGTGCGAGAATTTCAGGGAAAGAAT
TTATGGGGATTTCGAAGCAACTTCAGATGCTAACAACCCAATTTCCAAGTCATATCTT
GAGAAATTAAGGTCTATTTGCCCCCCAGTTGGGAAAGCTGCAGAGAACAACATAACA
GCAATGGACAATGTGACACCAGAGCTCTTTGACAACTCCTATTTCCACATCTTGATGA
GAGGAGAAGGGCTTATGAATTCAGACCAGGAATTGTATTCCAGCCTTCTGGGCATGG
AAACAAAGGCACTGGTGAAGAAATATGCTGCTGACCCACTTGCTTTCTTTCAGCAATT
CTCTGATTCCATGGTCAAGTTGGGAAATATCACCAACTCTGATAGCTTTTTCACTGGG
GAAGTTAGGAAAAACTGCAGGTTTATCAACACA

Cluster-19973.6

>CAAACCGCTCTCTCCCCGGCTACTGCACATCCCTCGCACACCGTTAGAGTTCGTTGT
GGTCAGATCAAAACGGATGCTAACAACGAGGATCGGTTTAGTAGAAGAGATGTTCTT
CAAGGTTTTGGTGGTACACTTGCTCTGGGATTAATGCTGAGCTCTGACCTAATGGTGG
AACCGGCTTATGCTGCTGATTTAATACAGCGCAGACAGCGATCTGAATTTTTATCAAA
TATCAAGGGCACCCTTTTTACAGCTATTAAGAAAAATCCAGACATCGTTCCCTCCCTT
TTAACATTAGCACTAAATGATGCTGTGACTTACGACAAGGCTACTAAAACTGGAGGG
CCAAATGCATCCATCCGCTTCAGTTCAGAGATTTCCAGACCTGAAAATGAGAAGTTTT
CTGCTGCTCTGAGTTTATTAGAGGAAGCAAAGAAGGAGATAGATTCGTATTCAAAGG
GTGGACCCATTTCCTACGCAGATCTTATCCAATTCGCAGCACAAAGTGCCGTTAAATC
TACCTTTCTAGCTTCAGCCATCAGAAAATGTGGTGGGAATGAAGAAAAGGGGAATTT
GTTATATACTGCGTATGGTTCCAATGGGCAGTGGGGCTTGTTCGACAGGCAATTTGGA
AGATCAGATGCTGAAGCGCCTGATCCTGAGGGAAGGGTTCCCGTCTGGGGGAAAGCT
AGCGTTCAGGAAATGAAAGAAAAGTTCTCTGCTATTGGATTTGGTCCCCGACAGCTA
GCTGTTTTGTCTGCATTTCTGGGTCCTGATCAGGCAGCAACTGAGGAACTTCTAGCCT
CAGATCCTGATGTTTTTCCATGGGTTCAAAAGTATCAACGTAGCCGTGAAACAGTATC
TCAGACTGATTATGAGGTCGATCTTATAACTACCCTGACAAAAATAAGTAGCTTGGG
ACAGCAGATAAATTACGAGGCTTACACCTATCCA

Cluster-21832.12599

>ATTTCTCACCTTTTTCTGAACCAGGGAAGGCCGAGTTCTTGTGTATCAAGTAGAGGA
TTCAATGCGGCGTCTCATCCGAAATGCTTAGCGTCGGACCCTGAGCAGTTGAAGAGT

GCTAAAGAAGATATCAAGGAGCTCCTGAAGACTACATTCTGCCATCCTATTTTGGTAC
GCCTGGGTTGGCATGATGCTGGAACTTACAATAAAAACATTGAGGAATGGCCGCAGA
GAGGTGGAGCTAATGGAAGTTTAAGATTTGATGTTGAGCTTGGACATGGTGCTAATG
CTGGTCTTGTCAATGCCTTGAAACTCATTGAGCCTATCAAAAAGAAGTATTCTAATGT
GACATATGCAGACCTATTCCAGCTGGCCAGTGCTACAGCTATCGAGGAGGCTGGGGG
ACCCAAAATCCCCATGAAGTATGGAAGGGTGGATGTTGTGGGGCCAGAGCAATGCCC
TGAAGAGGGAAGGCTTCCTGATGCTGGTCCTCCATCACCTGCTGCACATCTACGAGAT
GTTTTCTACAGAATGGGGTTGAATGACAAGGAAATAGTTGCACTATCTGGGGCACAC
ACGTTAGGGAGATCTAGGCCAGAACGCAGTGGTTGGGGCAAGCCAGAGACCAAATA
TACGAAAGATGGACCTGGTGCACCTGGTGGACAATCCTGGACCGTACAATGGTTGAA
GTTTGACAACTCATATTTCAAGGATATCAAAGAAAGGAGGGATGAAGAATTACTGGT
GTTGCCAACTGATGCTGCTCTCTTTGAAGATTCATCATTCAAGGTTTATGCTGAAAAA
TATGTTGATGATCAAGAAGCTTTCTTCAAAGATTATGCTGAAGCCCATGCCAAACTCA
GCAACCTTGGTGCTAAATTTGACCCTCCAGAGGGTATTGTGATCGATGATGCATCTTC
AAAACCTGCAGGAGAGAAGTTTGATGCCGCCAAATACTCATATGGGAAGGAT

Cluster-21832.17816
>ATGGTAAAGTGCTACCCTGCTGTTAGTGAGGAGTATCAAAAGGCCGTTGAGAAGGC
CAAGAGGAAGCTCAGAGGGCTCATCGCTGAGAAGAATTGTGCTCCTCTAATGCTTCG
TCTCGCATGGCACTCTGCTGGTACCTTTGACCATAAATCCAGGACTGGTGGTCCGTTT
GGAACCATGAGGTTCCCTAGTGAGCTGGCCCATGCTGCCAACAATGGCCTTGACATC
GCTATTAGGCTATTGGAGCCCATCAAGGAACAGTTCCCCATTCTCTCCTTTGCAGACT
TTTACCAGGTAATTTGTTTGATC

Cluster-21832.12605
>ATTTCTCACCTTTTTCTGAACCAGGGAAGGCCGAGTTCTTGTGTATCAAGTAGAGGA
TTCAATGCGGCGTCTCATCCGAAATGCTTAGCGTCGGACCCTGAGCAGTTGAAGAGT
GCTAAAGAAGATATCAAGGAGCTCCTGAAGACTACATTCTGCCATCCTATTTTGGTAC
GCCTGGGTTGGCATGATGCTGGAACTTACAATAAAAACATTGAGGAATGGCCGCAGA
GAGGTGGAGCTAATGGAAGTTTAAGATTTGATGTTGAGCTTGGACATGGTGCTAATG
CTGGTCTTGTCAATGCCTTGAAACTCATTGAGCCTATCAAAAAGAAGTATTCTAATGT
GACATATGCAGACCTATTCCAGCTGGCCAGTGCTACAGCTATCGAGGAGGCTGGGGG
ACCCAAAATCCCCATGAAGTATGGAAGGGTGGATGTTGTGGGGCCAGAGCAATGCCC
TGAAGAGGGAAGGCTTCCTGATGCTGGTCCTCCATCACCTGCTGCACATCTACGAGAT
GTTTTCTACAGAATGGGGTTGAATGACAAGGAAATAGTTGCACTATCTGGGGCACAC
ACGTTAGGGAGATCTAGGCCAGAACGCAGTGGTTGGGGCAAGCCAGAGACCAAATA
TACGAAAGATGGACCTGGTGCACCTGGTGGACAATCCTGGACCGTACAATGGTTGAA
GTTTGACAACTCATATTTCAAGGATATCAAAGAAAGGAGGGATGAAGAATTACTGGT
GTTGCCAACTGATGCTGCTCTCTTTGAAGATTCATCATTCAAGGTTTATGCTGAAAAA
TATGTTGATGATCAAGAAGCTTTCTTCAAAGATTATGCTGAAGCCCATGCCAAACTCA
GCAACCTTGGTGCTAAATTTGACCCTCCAGAGGGTATTGTGATCGATGATGCATCTTC
AAAACCTGCAGGAGAGAAGTTTGATGCCGCCAAATACTCATATGGGAAGAGAGAGCT
ATCAGACTCAATGAAGCAGAAGATTCGGGCAGAATACGAAGCTTTTGGTGGAAGTCC
AGATAAGCCTTTACCAACAAACTACTTCCTTAATATCATAGTTGTGATTGCTGTTTTGG

CCATTTTGACATCTCTGCTTGGAAAC
